# Preserved Self-Awareness following Extensive Bilateral Brain Damage to the Insula, Anterior Cingulate, and Medial Prefrontal Cortices

**DOI:** 10.1371/journal.pone.0038413

**Published:** 2012-08-22

**Authors:** Carissa L. Philippi, Justin S. Feinstein, Sahib S. Khalsa, Antonio Damasio, Daniel Tranel, Gregory Landini, Kenneth Williford, David Rudrauf

**Affiliations:** 1 Division of Behavioral Neurology and Cognitive Neuroscience, Department of Neurology, University of Iowa, Iowa City, Iowa, United States of America; 2 Semel Institute for Neuroscience and Human Behavior, University of California Los Angeles, Los Angeles, California, United States of America; 3 Brain and Creativity Institute and Dornsife Cognitive Neuroscience Imaging Center, University of Southern California, Los Angeles, California, United States of America; 4 Department of Philosophy, University of Iowa, Iowa City, Iowa, United States of America; 5 Department of Philosophy, University of Texas Arlington, Arlington, Texas, United States of America; Tokyo Metropolitan Institute of Medical Science, Japan

## Abstract

It has been proposed that self-awareness (SA), a multifaceted phenomenon central to human consciousness, depends critically on specific brain regions, namely the insular cortex, the anterior cingulate cortex (ACC), and the medial prefrontal cortex (mPFC). Such a proposal predicts that damage to these regions should disrupt or even abolish SA. We tested this prediction in a rare neurological patient with extensive bilateral brain damage encompassing the insula, ACC, mPFC, and the medial temporal lobes. In spite of severe amnesia, which partially affected his “autobiographical self”, the patient's SA remained fundamentally intact. His Core SA, including basic self-recognition and sense of self-agency, was preserved. His Extended SA and Introspective SA were also largely intact, as he has a stable self-concept and intact higher-order metacognitive abilities. The results suggest that the insular cortex, ACC and mPFC are not required for most aspects of SA. Our findings are compatible with the hypothesis that SA is likely to emerge from more distributed interactions among brain networks including those in the brainstem, thalamus, and posteromedial cortices.

## Introduction

Self-awareness (SA) is a complex, rich and integrated phenomenon of self-knowledge, which is central to consciousness and incorporates multiple components [1], [2], [3], [4], [5], [6], [7]. As a first approximation, one can distinguish several putative components of SA, which are hierarchically organized, and which might be partially dissociated, both functionally and neuroanatomically. Each component is in turn made up of multiple elements. The following description is meant to serve as a heuristic tool.

- *Core SA* is the most basic and fundamental component of SA and forms the foundation for all other components. It is grounded on the protoself, which includes “primordial feelings” of the living body [3], [8] and a preattentive, elementary form of self-consciousness [9]. On a moment to moment basis, Core SA generates a sense of personal agency and ownership over behavioral actions and sensory representations. Processes such as self-recognition and sentience require Core SA.
- *Extended SA* broadens Core SA to include an autobiographical self [3], [8], which involves an elaborate self-concept built upon a repository of autobiographical memories and representations of physical, affective and personality traits.
- *Introspective SA* relies on higher-order executive, attentional and metacognitive functions, which enable *introspection*, the ability to perform a more or less controlled *reflection* on one's own mental states, behaviors, and their consequences. Supported by memory and learning, introspective SA allows for the development of accurate knowledge about one's self, a capacity critical for efficient navigation of the social world [10], [11].

The study of the neural basis of SA has grown significantly over the past two decades [3], [4], [8], [12], [13], [14], [15], [16], [17]. Several recent theoretical frameworks produced partially overlapping hypotheses regarding the specific neural substrates of SA or various components of SA. One class of hypotheses emphasizes specific brain regions, which would play a central role in essential components of SA [18], [19], . Another class of hypotheses focuses on more distributed cortico-cortical and/or subcortical-cortical networks [4], [8], [16], [23], [24], [25], [26], [27].

Here we concentrate on the first class of hypotheses. Four main anatomical targets have been proposed based on a variety of neuroimaging findings: the insula, ACC, mPFC, and brainstem nuclei.

The *insular cortex* has been proposed as the critical substrate underlying SA in humans, necessary for interoceptive awareness [28], [29], [30], [31] and more generally for creating the “sentient self” or all of “human awareness” [20], [29]. On this hypothesis, the insula (especially the anterior insula) is presumed to represent the essential substrate for all components of SA (Core, Extended and Introspective).

The *ACC* has been implicated in functional neuroimaging studies of interoceptive and emotional awareness [3], [32], [33], [34], facial self-recognition [19], and more generally in the integration of our conscious experience [3], [35]. Moreover ACC activity is closely linked to the conscious monitoring of conflict [18], [36], [37], [38], and to the monitoring of self-related information underlying introspection [21]. Neuropsychological studies of patients with bilateral ACC damage provide evidence for the role of the ACC in emotion, motivation, and attention [39], [40], [41], [42], [43]. When ACC damage is combined with damage to the adjacent supplementary motor area, patients can manifest a profound state of akinetic mutism [42], [44]. Based on these findings, the ACC would play an important role in some aspects of both Core SA and Introspective SA.

The *mPFC* has been consistently implicated in self-referential processing [1], [21], [22], [45], [46], [47]. In functional imaging and lesion studies, the mPFC has also been associated with the autobiographical self [3], [48], [49], self-reflective thought [42], [50], and self-awareness and insight [14], [51], [52], as well as with the projection of the self into the future [53], and more generally with theory of mind [26], [54], [55]. Significant changes in personality have been consistently reported in patients with bilateral lesions to the ventral portions of the mPFC (vmPFC) [56], [57], [58], [59]. Besides personality changes, patients with lesions to the mPFC have impaired self-regulation in social settings, such as sharing too much personal information with a stranger [11]. Thus the mPFC would play a critical role in Extended SA and Introspective SA.

Finally, a specific set of nuclei in the *brainstem*, including the nucleus tractus solitarius, parabrachial nucleus, and the nuclei that compose the periacquaductal gray, has been hypothesized as the neural basis for the “primordial feelings” of the living body, an essential contributor to Core SA that sets the foundation for both Extended and Introspective forms of SA [4], [8]. Of note, these nuclei are not those belonging to the ascending reticular activating system, which traditionally have been related to other aspects of consciousness, namely the processes of wakefulness and attention [12].

**Figure 1:**
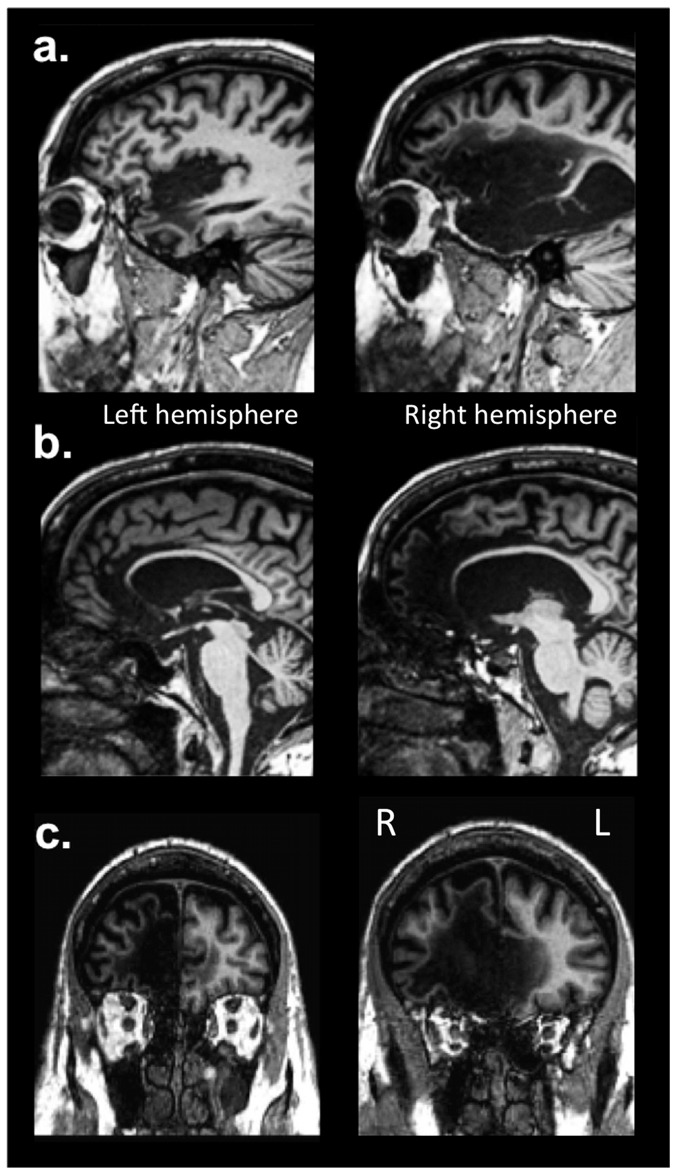
T1-weighted MRI scans of R's lesion. (a) Sagittal images highlighting damage to the insular cortex, (b) sagittal images highlighting damage to the ACC, and (c) coronal images highlighting damage to the mPFC. All three structures are extensively damaged, bilaterally, in R's brain.

**Figure 2:**
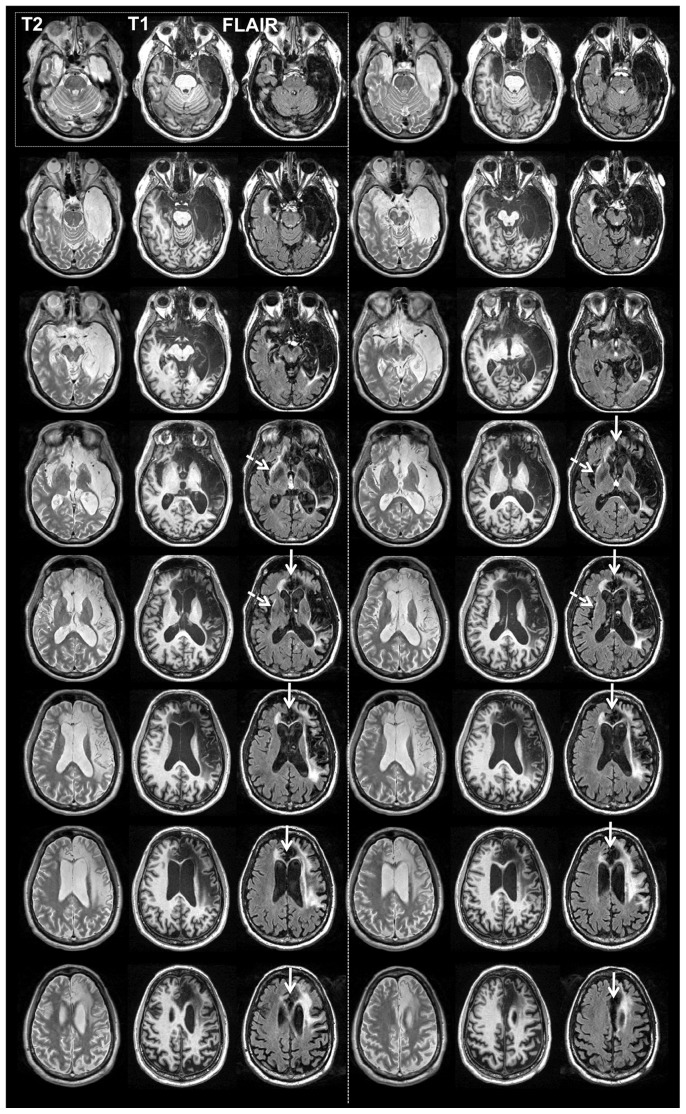
Structural imaging. Series of axial slices organized in a ventral-to-dorsal direction (ventral-most = top left; dorsal-most = bottom right). Slices are grouped in series of three, corresponding respectively to T2-weighted, T1-weighted and FLAIR imaging sequences of the same slice in the brain. The ventral-most set is in the top-left; next is the top-right; next is the second row-left, next is the second row-right, etc. Slices are sampled every 4 mm. Slices are in neurological convention (left side of image = left hemisphere; right side of image = right hemisphere). Dashed white arrows point to areas of abnormality detected on the FLAIR scan within the region of the left anterior insula. Continuous white arrows point to the mPFC/ACC region, highlighting the extensive bilateral destruction of these regions.

In this study, we assessed SA in a rare neurological patient, R (referred to as ‘Roger’ in previous publications), who has extensive bilateral damage to the insula, ACC and mPFC [60] (Figures 1, 2). R's case presented a unique opportunity to: 1) evaluate the critical role of the insula, ACC and mPFC in SA, and 2) assess the profile of SA at multiple levels in a single patient using a broad array of tasks. We reasoned that if any of the structures that are damaged in this patient are indeed critical for the different aspects of SA implicated by the hypotheses reviewed above – i.e., insula, ACC, mPFC - the patient should show clear disruptions of the corresponding functions. Conversely, if these structures are not critical, R should show largely preserved SA.

## Materials and Methods

The study was approved by the Institutional Review Board at the University of Iowa. Written informed consent was obtained from the patient and his family to conduct this study.

### Participant

Patient, R is a 57 year old, right-handed, college-educated, male, whose brain was damaged in 1980 following a severe episode of herpes simplex encephalitis. His brain damage is bilateral, more extensive on the right, and encompasses the target regions of the hypotheses under scrutiny: the insular cortex, the ACC, and the mPFC (Figures 1, 2). The damage extends to the basal forebrain and the entire medial temporal lobe, including the amygdala and hippocampus bilaterally. The white matter is extensively damaged, in particular on the right, from the frontal and temporal poles to the anterior inferior parietal lobule. The damage largely spares the brainstem, thalamus, hypothalamus, basal ganglia, and most posterior cortical structures in the occipital and parietal lobes. R presents with a profound anterograde amnesia and a temporally graded retrograde amnesia that is most apparent during the 10 years preceding the onset of his brain damage. He is also ageusic and anosmic. Remarkably, R exhibits intelligence within the normal range and a largely normal neuropsychological profile (see [60] for further details on R's case).

### Neuroimaging

Magnetic Resonance Imaging (MRI) data were acquired with a 3.0-Tesla Siemens Trio MRI scanner using a 12-channel head coil. Three T1-weighted MRI scans were acquired (magnetization prepared rapid gradient echo, MPRAGE, AC-PC aligned coronal acquisition; time to repetition, TR = 2,530 ms; echo time, TE = 3 ms; inversion time, TI = 800 ms; flip angle = 10 degrees; field of view, FOV = 256×256 mm^2^; slice thickness = 1 mm). The images were bias field corrected and registered together with a rigid body transformation and a sinc interpolation (Automated Image Registration [AIR] 3.08). The three scans were then averaged together in order to reduce motion artifacts, increase the signal-to-noise ratio, and enhance the contrast-to-noise ratio between gray and white matter. T2-weighted images were acquired using a turbo spin-echo sequence with the following parameters: FOV = 240×240 mm^2^; matrix = 256×256; TE = 14 ms; TR = 6350 ms; slice thickness = 2 mm; number of excitations, NEX = 2; bandwidth, BW = 315 Hz/Pixel; Echo Train Length = 9, and Integral Parallel Imaging Technique, IPAT = 2. Fluid Attenuated Inversion Recover (FLAIR) data were acquired using the following parameters: FOV = 240×240 mm^2^; matrix = 256×256; TI = 2500 ms; TE = 115 ms; TR = 9120 ms; NEX = 1; slice thickness = 2 mm; BW = 241 Hz/pixel, Echo Train Length = 23, and IPAT = 2. Diffusion Tensor Imaging (DTI) data were acquired using the following parameters: voxel size = 2×2×2 mm^3^; FOV = 256×256 mm^2^; matrix = 128×128; NEX = 1; TE = 100 ms; TR = 8,500 ms; slice thickness = 2 mm; gap = 0; number of directions = 30; B value = 1000; and BW = 1396 Hz/pixel. DTI data were processed first using 3 d Slicer [61] to convert Digital Imaging and Communications in Medicine (DICOM) files into Nearly Raw Raster Data (NRRD) format and access accurate gradient direction information, and then using the Functional Magnetic Resonance Imaging of the Brain (FMRIB) Diffusion Toolbox from FSL [62], [63] to extract measures of fractional anisotropy (FA). The FA data were then registered to the FMRIB58 FA atlas provided in FMRIB Software Library (FSL) using FMRIB's Linear Image Registration Tool (FLIRT) after smoothing using a Gaussian kernel (Full-Width at Half-Maximum = 2 mm^3^) in order to provide a basis for comparison.

Volumetric quantification of potentially spared tissue within R's insula and ACC was also performed using T1-weighted images. Manual tracings of the small islands of remaining tissue found within the left anterior insula, left posterior insula, and left dorsal ACC were conducted using standardized tracing methods for the insula [64] and ACC [65], [66]. These estimated volumes were compared to normative volumes for both the insula [64] and ACC [65] in order to derive a percentage of estimated preserved tissue in the regions of interest.

Finally, basic functional MRI (fMRI) data in R was examined in order to: (1) ascertain the properties of his blood oxygenation level dependent [67] response, and (2) examine the degree of functional connectivity within different brain networks, including regions with potentially spared tissue (e.g., the left anterior insula). Since we did not collect actual resting state data, we instead estimated functional connectivity from residualized fMRI data obtained using a simple sensorimotor block-design protocol containing interspersed blocks of passive fixation. A similar approach has been shown to provide results that are qualitatively, and to a large extent quantitatively, comparable to functional connectivity derived from actual resting state data [68]. These data are used in order to provide some further qualitative analysis regarding the functional status of R's left anterior insula, in addition to structures within the default network.

We used the Phase I sensorimotor protocol from the functional Biomedical Informatics Research Network (fBIRN) sequence [69]. The sensorimotor task consisted of a block design with block durations of 15 s on/off, including 8 complete on/off cycles for a total scan time of 240 s. During on-blocks, the participant performed a finger tapping task while listening to a 3 Hz audio cue and while watching a 3 Hz flashing checkerboard. Tones were 333 ms long. The participant was asked to tap each finger, one finger at a time (from the index finger to the pinky finger) in-synchrony with the tones using both hands simultaneously. During off-blocks there was passive visual fixation of a cross during rest and no finger tapping. The protocol was delivered using E-Prime (http://www.pstnet.com/eprime.cfm). We acquired two runs of this protocol.

Echo Planar Imaging (EPI) gradient-echo BOLD data were acquired during the sensorimotor protocol (matrix = 64×64; TE = 40 ms; TR = 3000 ms; voxel size = 3.5×3.5×4 mm3; number of slices = 35). B0 field maps were also acquired for unwrapping. Registration of the EPI-BOLD data to T1-weighted images was performed using FLIRT. The EPI data were first registered to the 4th volume of the first run for motion correction, time-corrected and despiked using Analysis of Functional NeuroImages (AFNI) [70], concatenated, linearly detrended, band-passed filtered (0.009–0.08 Hz), and then spatially smoothed using a 5 mm Gaussian kernel.

The sensorimotor protocol was then analyzed using a standard hemodynamic response function convolved with the task block-design function and entered in a general linear model, including as additional covariates the 6 motion parameters and their time derivatives from the motion correction registration phase. After pre-processing, all analyses were completed using in-house scripts in Matlab.

Functional connectivity analysis on the residualized fBIRN data was performed using the same basic preprocessing pipeline as the sensorimotor task. In addition, the general linear model was used to extract residuals after covarying out the 6 motion parameters and their time derivatives as well as the average BOLD time courses for the following regressors: white matter voxels, cerebrospinal fluid (CSF) voxels within the ventricles, and the global brain signal. We then used a seed-based region of interest [71] analysis of functional connectivity (based on [72]). Seeds were placed in the following regions: the left lingual gyrus (as a control region to characterize functional connectivity within the visual cortices), the left posterior cingulate (in order to assess for the presence of residual functional connectivity between spared regions of the Default Mode Network), and in the potentially spared tissue of the left anterior insula (in order to explore the functional status of this region in comparison to a previous study [73] documenting patterns of functional connectivity with the insular cortex). The tracing of the seed regions was done using both the average EPI image registered to the T1-weighted space and the T1-weighted image registered to the EPI space, in order to help with identifying voxels corresponding to the island of spared tissue in the anterior insula. We kept a smoothing kernel for all analyses sufficiently small (5 mm) to minimize contamination of the BOLD signal from this island of insular tissues with signal coming from surrounding areas. For comparison, we used the same kernel for the analyses using seeds in other locations. For each seed region, the residual time courses were averaged across voxels. The average was used as a reference time course in a voxelwise functional connectivity analysis. Correlation coefficients between the reference time course and all other intracerebral voxels were calculated and used as a measure of functional connectivity.

### Stimuli and procedures

We administered an extensive set of established and novel tasks assessing different aspects of SA, covering essential components of Core, Extended and Introspective SA. This included tests of basic self-recognition, self-agency, and self-concept. We also conducted a self-awareness interview probing the patient's metacognitive, reflective and introspective abilities, domains that are difficult to measure with standard laboratory tasks. The specific tasks were chosen based on several criteria: (1) we attempted to use paradigms which have been previously validated to measure SA or one of its components, (2) we created some tasks specifically for R in order to further tap into certain components of his condition, and (3) we tried to select tasks that had a high level of face validity in terms of their relationship to SA. The tasks described below are grouped under the main component of SA that they are testing (Core, Extended or Introspective); however, we note that many tasks rely on a combination of different components of SA.

Basic self-recognition tasks addressing aspects of Core SA focused on the ability to recognize oneself as separate from the environment. Basic self-agency tasks addressing aspects of Core SA focused on the ability to distinguish between self-generated and other-generated action, probing the basic knowledge that one is the causal agent of an action (i.e., the sense of self-agency), a central component of Core SA [81]. Here we use two tasks to probe two types of self-agency: (1) the feeling of agency and (2) the judgment of agency [82]. The agency tasks used in this study can be described using these distinctions, with the “Tickle Test” assessing the feeling of agency and the “Self-Agency Judgment Task” assessing the judgment of agency.

#### Core SA: Basic Self-Recognition Tasks, Mirror Self-Recognition

Visual recognition of one's self in a mirror has been a gold-standard probe for assessing SA in animals [74], [75], [76]. Mirror self-recognition is present early in human development as most infants learn to identify their reflection at around 18 months of age [77]. We assessed mirror self-recognition with a protocol adapted from a previous study in children with autism [78]. *Materials*: one 12″×12″ mirror mounted in a black frame, one frame holder, facial tissues, and black eye shadow. Based on pilot trials, the eye shadow was selected to assure it would not be perceptible (e.g., as a cold sensation on the skin) upon application. *Protocol*: We first placed a mirror on a table in front of R for approximately 1 minute to assure that he could see himself and to collect any baseline responses. Then, R was directed away from the mirror to participate in a distracter task (counting circles in a figure). During this task, black eye shadow hidden in a facial tissue was inconspicuously rubbed on R's nose by the experimenter (as if brushing something off his nose). After a 15 minute delay, R was brought back to the mirror and all reactions were audio and video recorded for subsequent coding. Note, the 15 minute delay was sufficient to ensure that R would not remember any explicit details about the application of the eye shadow given his severe anterograde amnesia.

#### Core SA: Basic Self-Recognition Tasks, Self-Recognition in Photographs

In order to assess self-recognition in ecological conditions (including context and extra-facial features), 16 digital photographs of R were used. These photos varied in the following dimensions: (1) R's age (ranging from 5–55 years), (2) number of people (ranging from 1 to 6), and (3) R's familiarity with those in the photos (family members, familiar friends from before brain injury, or familiar friends from after the brain injury). A second set of 16 digital photographs (foils) without R were matched to the target photos for number of people, gender, and background appearance (i.e., setting). Moreover, some of the foils contained individuals who looked very similar to R. The two sets of photos (32 total photos) were presented randomly to R, one photo at a time, with no time limit. For each photo R was asked two questions: 1) “Do you see yourself in this photo?”, and 2) “If so, could you point to yourself in the photo?” This task was administered twice on consecutive days.

#### Core SA: Basic Self-Recognition Tasks, Self-Face Recognition

A variety of self-face recognition tasks have been used as measures of SA in normal, neuropsychological, and neuropsychiatric populations [79]. In a typical visual self-face recognition task, subjects are asked to identify pictures of their own faces, usually devoid of both contextual information and extrafacial features. In the current study, we administered a basic self-face recognition task similar to those used in previous studies (e.g., [80] – Experiments 1 and 2). We used the same target pictures as those in the *Self-Recognition in Photographs* task. All faces were individually cropped from the photographs and extrafacial features were removed (e.g., clothing, hair, etc). Cropped faces were then placed on a black background and pictures were equated for dimension and resolution. The self-face recognition task consisted of 4 different conditions with 48 total stimuli: 1) self (pictures of R; n = 16), 2) family (pictures of his family members; n = 8), 3) familiar others (pictures of experimenters he has worked with, but that he should not remember given his anterograde memory impairment; n = 8), and 4) unfamiliar others (pictures of unfamiliar people; n = 16). For each photo, R had to respond to the following questions in order to assess recognition and familiarity of the stimuli: 1) “Is the face familiar, not familiar, or is it you?”, 2) “Rate the familiarity of the face from 0 ‘not at all familiar’ to 10 ‘extremely familiar’”, 3) “Rate the face on a scale from 0 ‘definitely not me’ to 10 ‘definitely me’”. Stimuli were randomly presented and remained on the screen until the participant responded to all questions.

#### Core SA: Basic Self-Agency Tasks, Tickle Test

The ability to discriminate between self initiated touch as compared with touch initiated by an external source was measured with a standard paradigm, the tickle task [83]. A study by Blakemore and colleagues showed that self-administered tickle was perceived as less ticklish than externally-administered tickle [84]. The tickle task has been used as an implicit measure of intact self-agency and self-monitoring in both normal healthy subjects and in psychiatric conditions (e.g., schizophrenia) [83], [85]. *Procedure*: The participant's ticklishness was measured with a self-report questionnaire and two tickle administration conditions (self and experimenter). Six different body parts were probed in random order: palm of the hands, bottom of the feet, belly, sides of the torso by the ribs, knees, and armpits. During the tickle task, the participant was seated in a chair. In both the self and experimenter conditions, tactile stimulation (tickling) was administered to both sides of the body using the fingertips. Each body part (n = 6) was tickled using the fingertips (by either R or the experimenter) for approximately 5–10 seconds. Following each tickle administration, the participant was immediately asked to rate the perceived ticklishness on a scale of 1 (not at all) to 10 (extremely ticklish). The instructions were repeated for both the experimenter-administered and self-administered tickle conditions.

#### Core SA: Basic Self-Agency Tasks, Self-Agency Judgment Task

The ability to determine whether one is responsible for one's own actions was assessed in a self-agency judgment task. This task was modeled after previous studies of self-agency [86]. Recent functional neuroimaging studies of self-attribution of agency have found distinct neuroanatomical dissociations between self- and other-attribution of agency in the anterior insula and inferior parietal lobule, respectively [86], [87]. Whereas activity in the right inferior parietal lobule was associated with the experience of another performing an action, activity in the insula was associated with experience of self-agency [86], [87]. *Procedure*: Using a mouse, the participant had to move a blue box presented at the center of the screen onto a green box presented randomly at one corner of the screen. The participant had 10–seconds to move the blue box into the green box. After each trial, the participants were immediately asked how much control they felt they had over the blue box. Responses were collected using a visual analogue scale ranging from 0 “no control” to 100 “complete control”. Unbeknownst to the participants, perceived control was manipulated parametrically by varying the proportion of time the participants had control over the blue box during a trial, ranging from 0 to 100 percent of the time. There were 50 randomly presented trials, 10 for each of the five different temporal conditions designed to vary perceived control (0, .30, .75, .9, 1.0).

#### Extended and Introspective SA: Basic Self-Concept tasks, Self-Consciousness Scale Revised (SCSR)

We assessed R's level of self-consciousness using a well-established self-report questionnaire, the Self-Consciousness Scale Revised (SCSR) [88]. The questionnaire was designed to measure personal insight, self-focused attention, and the endorsement of behaviors and thought processes characteristic of different types of SA. Some example items include, “Before I leave my house, I check how I look” and “It's hard for me to work when someone is watching me”. Ratings are provided on a 4-point rating scale ranging from 0 (not at all like me) to 3 (a lot like me). Separate scores were computed for each of the three subscales of the SCSR: public self-consciousness, private self-consciousness, and social anxiety. In order to interpret R's SCSR scores, we used normative data reported in a previous study establishing the psychometric properties of the SCSR [88].

#### Extended and Introspective SA: Basic Self-Concept tasks, Self-Related Positivity Bias Task

Knowledge of one's own personality traits, and more generally self-concept, is not always accurate. In fact, there is evidence to suggest that most people view themselves in a more positive light than they view others. This positivity bias is the tendency to accentuate the positive when evaluating self-related information [89]. When measured using trait ratings, most people endorse more positive traits as being self-descriptive as compared to negative traits. We used a trait adjective rating paradigm [90] in order to assess whether R also exhibited a positivity bias when reflecting on his self-concept. *Procedure*: A set of 270 trait adjectives (e.g., “genuine”, “mean”) were selected (135-negative traits and 135-positive traits as normed in [91]. During the task the participant judged each personality trait based on the degree of self-relevance, indexed by a 4-point rating scale (1 = “not at all like me” to 4 = “very much like me”). On each trial, a trait adjective was presented for 1250 ms followed by presentation of a fixation cross for 750 ms. Responses were recorded on a keyboard by pressing buttons 1, 2, 3, or 4. Detailed instructions and practice trials were given before the experimental task runs in order to ensure that the subject understood and was comfortable with the task. Trial order was randomized. Seven gender- and age-matched comparison subjects also completed the task.

#### Extended and Introspective SA: Basic Self-Concept tasks, Big-Five Inventory (BFI)

R's personality ratings were collected using a well-validated personality questionnaire, the BFI [92]. The BFI is a self-report 44-item questionnaire designed to evaluate the big five personality dimensions (extraversion, agreeableness, conscientiousness, neuroticism, and openness). We administered the BFI to R at two separate times (baseline and 1-year follow up) in order to investigate the stability of his self-concept over time. We also had R's sister and mother rate his personality using the same questionnaire, as a proxy for objectively measuring the accuracy of his self-concept. Finally, we compared R's personality ratings on the BFI to a sample of 796 males [93], all in their 50's, in order to provide a general sense of whether any of his self-reported traits are outside of the normal range. Of note, R has previously completed the Minnesota Multiphasic Personality Inventory Version 2 (MMPI-2) and has been found to have a completely normal profile [60].

#### Extended and Introspective SA: Anosognosia tasks, Basic questionnaires

R's awareness of his behavioral and neuropsychological deficits was measured using a well-established 30-item self-report measure, the Patient Competency Rating Scale (PCRS) [94]. Thirteen additional questions were added targeting a variety of specific deficits (e.g., anosmia). All questions were completed by R twice, on consecutive days. A collateral version of this questionnaire was completed separately by two of the authors (JSF and DT) who have worked extensively with R for many years and have had numerous opportunities to observe the depth and scope of his impairments. Difference scores were computed for each question by taking the average of R's two responses and subtracting the average of the two collaterals. Based on this computation, larger differences reflected greater levels of anosognosia.

#### Extended and Introspective SA: Anosognosia tasks, Smell Test

To assess the depth of R's specific anosognosia for his anosmia (inability to smell), we conducted a smell test aimed at directly exposing R to his impairment. Procedure: There were 4 items used during the smell test: 2 items with a strong scent (onion and lemon), and 2 odorless items (tape and a brand new plastic pipe cleaner). R was told before the task began that some items would smell and others would not. There were 2 different conditions (blindfold on, blindfold off) in which the item was placed in front of the participant's nose, and he was asked to sniff. After about 30 seconds of repeated inhalation with the blindfold on, the participant was asked two questions: 1) “What do you think this is?” and 2) “What does it smell like?” The blindfold was then immediately removed and R was asked further questions to encourage reflection on his impairment and appraisal of his awareness (e.g., “Why do you think you were unable to smell the lemon with the blindfold on?”). The main goal of this test was to determine whether R would acknowledge that he has a problem with his ability to smell following the realization that he could not smell anything with the blindfold on.

#### Extended and Introspective SA: Metacognitive and Conceptual Processing, SA interview

R was asked a series of questions during a comprehensive interview designed to probe more conceptual and metacognitive aspects of SA. The interview was roughly divided into questions probing a basic understanding of concepts (e.g., How would you define consciousness?) to questions probing more introspective and metacognitive aspects of SA (e.g., Do you have a sense of self?).

## Results

### Imaging results

**Figure 3:**
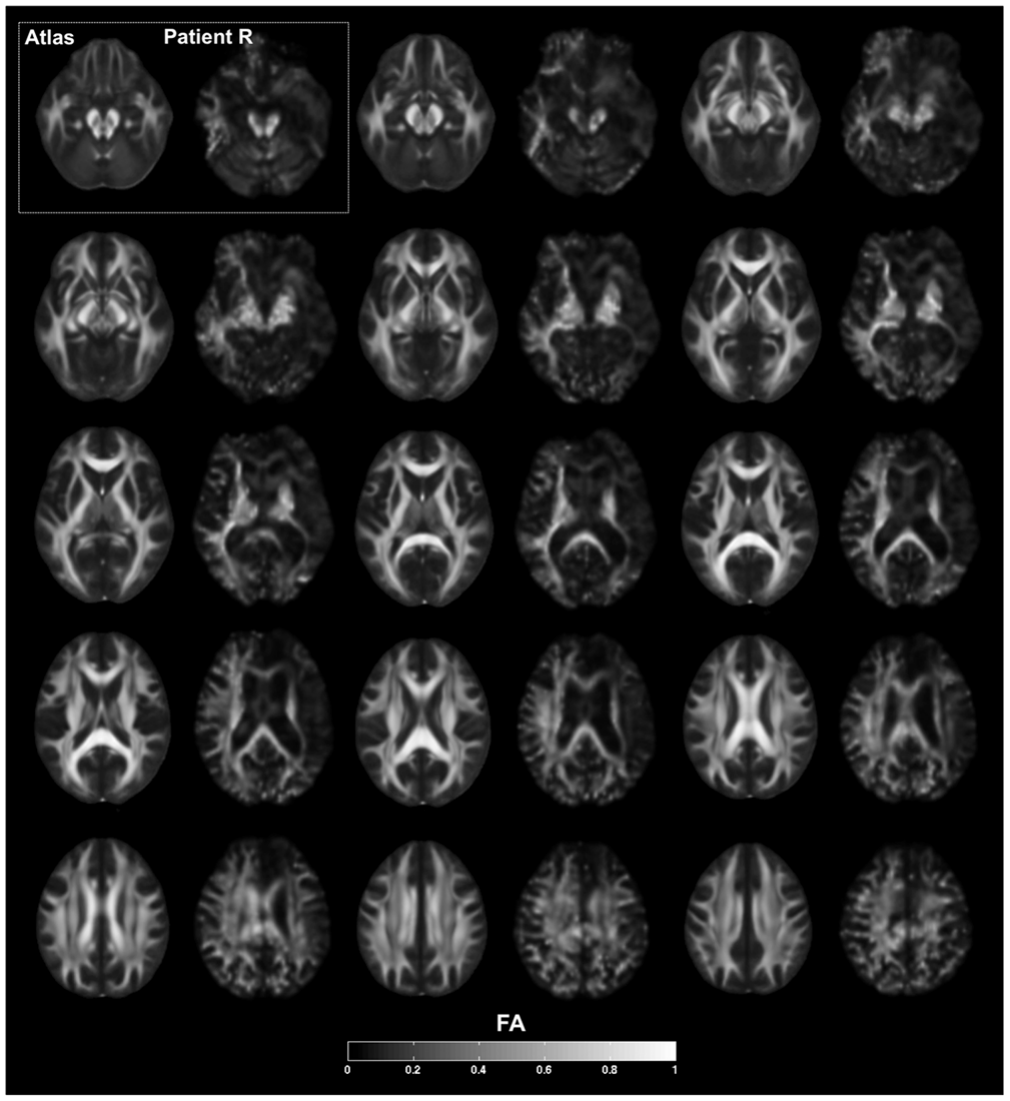
Fractional anisotropy. Series of axial slices organized in a ventral-to-dorsal direction (ventral-most = top left; dorsal-most = bottom right, as in Figure 2) showing fractional anisotropy (FA) from DTI. Slices are grouped in series of two, corresponding respectively to average values from the FMRIB58 FA atlas and R's brain. Slices are sampled every 4 mm. Slices are in neurological convention, with the left hemisphere on the left. The legend at the bottom of the figure shows the grayscale coding of the degree of FA, ranging from 0 to 1. The results indicate a diffuse and global relative reduction in FA, indicative of extensive white matter pathology and widespread disconnection, especially in the frontal and temporal lobes. There is greater degree of damage in the right hemisphere.

A detailed description of R's brain damage has been previously reported [60]. Here, we highlight the main findings with regard to the three regions of interest relevant to the current study: the insula, ACC, and mPFC. Consistent with R's T1-weighted MRI scans (Figure 1–2), both the FLAIR and T2-weighted images (Figure 2) confirmed the presence of extensive and bilateral tissue abnormality throughout the insula, ACC, and mPFC. The DTI images, when compared to the atlas, revealed a diffuse and global relative reduction in FA, indicative of extensive white matter pathology and widespread disconnection, especially in the frontal and temporal lobes (Figure 3). There was a greater emphasis of damage in the right hemisphere. With regard to the mPFC, the T1-weighted images highlight a complete destruction of the right mPFC, and a partial destruction of the left mPFC, with some sparing of the most ventral and dorsal sectors. However, upon further inspection of the FLAIR and T2-weighted images (Figures 2), the signal from the left mPFC is highly abnormal, including in regions that appeared to be spared on the T1-weighted images.

The volumetric analysis quantified the percentage of potentially spared tissue in R's insula and ACC as detected on T1-weighted images. Utilizing standardized manual tracing procedures [64], [65] we estimate that R has 0% of his right anterior insula remaining and 0% of his right posterior insula remaining. Likewise, we estimate that he has 0% of his right anterior cingulate remaining. In the left hemisphere, we detected two islands of potentially spared tissue located in dorsal sectors of the anterior and posterior limits of the insula. We estimate that R has 26.8% (±1 standard deviation, SD: 20.6%–32.0%) of his left anterior insula remaining and 12.3% (±1 SD: 10.5%–14.8%) remaining in his left posterior insula. When combined, this equates to approximately 22% of his left insula. In the left ACC, we detected a small region of tissue in the most posterior sector of Brodmann area (BA) 24 that we estimate to be less than 1% of his left ACC. Of note, the more dorsal and posterior aspects of BA 32 appear to be spared in R's left hemisphere. However, this remaining tissue is dorsal to the paracingulate sulcus, and is therefore considered part of the paracingulate cortex and not computed in volumetric analyses of the ACC [66]. In total, the volumetric analyses indicate that R is missing ∼90% of his insular cortex, bilaterally, and ∼99% of his ACC, bilaterally.

Careful inspection of the FLAIR and T2-weighted images (Figure 2), indicates that the potentially spared tissue in R's left anterior and posterior insula manifests is abnormal. The only exception is a small island of tissue in the most dorsal aspect of the left anterior insula. This raises the question about whether the remaining island of tissue in the left dorsal anterior insula (equating to less than 10% of total insula volume) is still “functional” in a way that could compensate for all the missing tissue.

**Figure 4:**
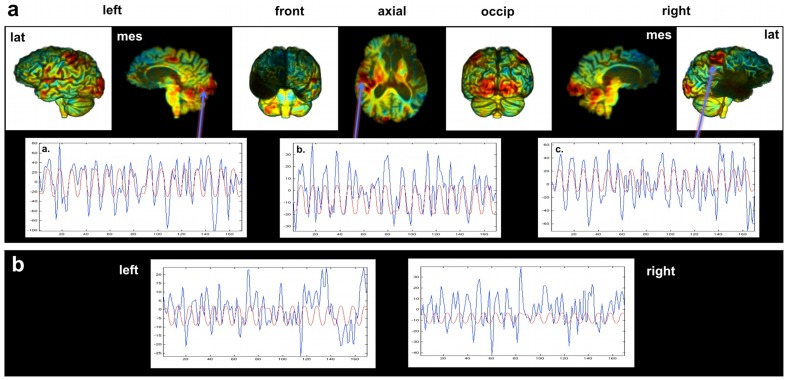
R's BOLD response during the sensorimotor fMRI paradigm. **a.** Upper tier – Unthresholded images showing t-values using a color code that goes from blue to red (ranging from −7 to +7 in t-values). Lower tier – R's actual BOLD response (blue) mapped on the same graph as his predicted BOLD response (red) for voxels in: (a) primary visual cortex, (b) left primary auditory cortex, and (c) right primary somatomotor cortex. The observed and predicted time courses are clearly in phase, indicating normal BOLD response in these regions. **b.** R's actual BOLD response (blue) mapped on the same graph as his predicted BOLD response (red) for voxels in the left and right anterior insula. The observed and predicted time courses are not in phase (the presence of oscillations in similar frequency ranges is driven by the bandpass filter). Thus, signal from both the left and right anterior insula voxels did not correlate with the predicted BOLD response, indicating that signal emanating from these regions during the task is likely noise-related.

One way to probe the functional status of this tissue with the available data is by examining its functional connectivity during fMRI. First though, it is important to establish whether R manifests a normal BOLD response. While performing a standard sensorimotor task, R displayed the expected pattern of BOLD responses (Figure 4). He showed activation in the hand region of the somatomotor and somatosensory cortices, bilaterally (related to finger tapping), in the primary and secondary visual cortices, bilaterally (related to visual stimulation), and in the primary auditory cortex of the left hemisphere (in response to the tones). Of note, he did not show any BOLD response to the protocol in regions that appeared damaged on structural MRI data, including right Heschl's gyrus. Figure 4a shows the time course of his BOLD responses in the activated regions in parallel to the predicted BOLD response based on the stimulus function. In all target regions there is a close correspondence between the predicted and actual BOLD response (Figure 4a). Of note, there were no significant clusters of activity in the left anterior insula (although anterior insula activation was not necessarily expected during the fBIRN sensorimotor protocol). Nevertheless, we used voxels in the island of the left anterior insula and in the space where the anterior insula was expected to be on the right, for qualitative comparison (Figure 4b). Signal from both the left and right insula did not show particular correlations with the predicted BOLD response, indicating that signal emanating from these regions during the task is likely noise-related.

**Figure 5:**
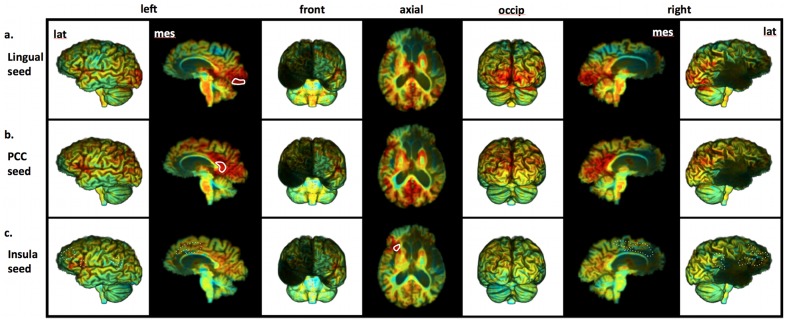
R's functional connectivity results. Unthresholded images showing correlation values using a color code that goes from blue to red (ranging from −0.9 to 0.9). Seed regions used for each analysis are delineated with a solid white line. (a) Results for the left lingual gyrus seed region, showing robust functional connectivity with the rest of early visual cortices in both hemispheres. (b) Results for the left posterior cingulate cortex seed region, showing widespread functional connectivity throughout the posteromedial and lateral parietal cortices, but essentially no signs of connectivity with the mPFC. (c) Results for the left dorsal anterior insula seed region, which failed to show any evidence of functional connectivity with regions (see dashed white lines) that have been previously shown to be functionally connected with the anterior insula [73].

Turning to the functional connectivity results, we first examined “control” (undamaged) regions to establish whether R's brain shows normal functional connectivity in undamaged areas. When a seed was placed in the left lingual gyrus, R showed robust functional connectivity with the rest of early visual cortices in both hemispheres (Figure 5a). Likewise, a seed placed in the left posterior cingulate cortex (a region known to be part of the “Default Network”) revealed widespread functional connectivity throughout the posteromedial and lateral parietal cortices (Figure 5b), with essentially no signs of connectivity with the mPFC (the only exception being a small region of connectivity with the most dorsal aspect of the left mPFC).

What about the potentially spared tissue of his left dorsal anterior insula? When a seed was placed in this area, there was no indication of normal functional connectivity (Figure 5c). In particular, there was no evidence of functional connectivity with regions that have previously been shown to be reliably functionally connected with the left anterior insula (e.g., the left dorsolateral prefrontal cortex and the supplementary motor area, both of which are intact in R's brain) [73]. Overall, the functional connectivity results suggest that the small regions of potentially spared tissue in R's left dorsal anterior insula and left mPFC are not functioning normally – in fact, a likely possibility is that this tissue is not functioning at all.

### Behavioral results

#### Core SA: Basic Self-Recognition Tasks, Mirror Self-Recognition

R displayed intact self-recognition as measured by the mirror task. When the makeup was applied to his face in a facial tissue, R barely blinked an eye and seamlessly continued to tell jokes and share stories with the experimenter during and after the distractor task. During the 15-minute delay, he did not show any indication that he was aware of the presence of the makeup (e.g., by touching his nose). Instead R remained engaged in the conversation and was attentive to his external environment. Upon returning to his seat in front of the mirror, he readily noticed and wiped the makeup off his nose. He also commented, “I wonder how that got there?” R then moved on to other grooming behaviors while looking in the mirror (e.g., fixing his hair). He also demonstrated self-recognition on numerous occasions throughout the multiple testing sessions upon spontaneously seeing his reflection, making comments such as “that's me” or “I can see myself”.

#### Core SA: Basic Self-Recognition Tasks, Self-Recognition in Photographs

**Figure 6:**
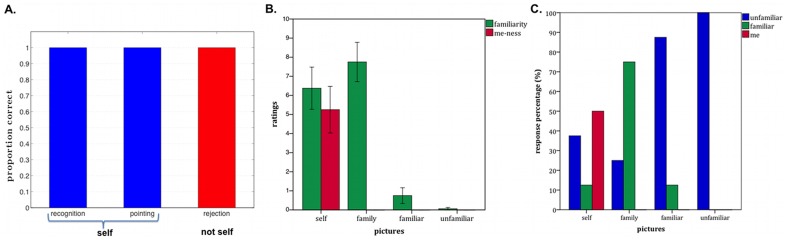
Self-recognition from pictures. A. Self-recognition in photographs (not cropped and with context): The proportion of correct self-recognition (Do you see yourself in this photo?) and pointing (If so, could you point to yourself in the photo?) was 100% accurate. There were no false positive ratings; all pictures without R were correctly rejected (red). B. Self-face recognition (cropped pictures without extrafacial features or context): Ratings of “me-ness” (red) and familiarity (green) ranging from 0 (not at all) to 10 (extremely) for pictures of faces of R, family members, familiar persons, and unfamiliar persons. Error bars are standard errors. C. Forced choice classification as “me,” “familiar” or “unfamiliar” for the same conditions as in B. The results in 6B and 6C show that R had some difficulty in recognizing himself in the same set of pictures when they were altered to exclude all extrafacial features. However, he never rated a picture of another person as himself, and he correctly recognized 75% of the faces of his family members.

In the self-recognition in photographs task, R performed perfectly, with 100% accuracy across all 32 photos on both days the task was administered (Figure 6a). He immediately pointed himself out in every photo, and never incorrectly said he was someone else in any of the lures, including photographs depicting individuals with a similar appearance to him. Anecdotally, he displayed spontaneous self-referential processing, occasionally commenting on his appearance in the photo and comparing it to his present appearance (e.g., comparing the attire he was wearing in the photo to the attire he was wearing during the day of testing). Given his profound anterograde amnesia, R's perfect performance on this task was remarkable as he was able to correctly identify himself in pictures taken decades after the onset of his amnesia.

#### Core SA: Basic Self-Recognition Tasks, Self-Face Recognition

Despite having intact self-recognition in unaltered photographs taken during all periods of his lifetime, R had some difficulty in recognizing himself in the same set of pictures when they were altered to exclude all extrafacial features (Figure 6b,c). Under these conditions, he only correctly identified himself (i.e., face stimuli that were rated as “me”) in 50% of the photos of himself (8/16), and even rated some pictures of himself as completely unfamiliar. However, he never rated a picture of another person as himself. R correctly recognized 75% of the faces of his family members (6/8). Both of the pictures he missed in this set of family member's faces were of his sister. Consistent with his anterograde memory deficits, he rated 88% of the faces of people he met after his brain injury (e.g., researchers who he has met on numerous occasions) as unfamiliar (7/8). Of note, he never rated any strangers as being familiar.

#### Core SA: Basic Self-Agency Tasks, Tickle Test

**Figure 7:**
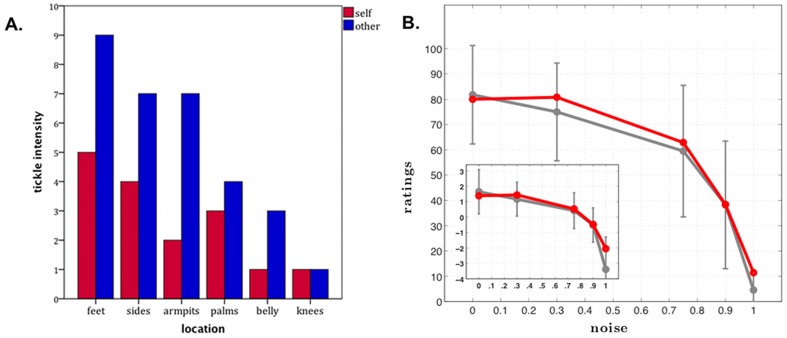
Self-Agency. A. Tickle test results. Tickle intensity ratings (y-axis) are plotted as a function of the body part being tickled (x-axis). Tickle condition is color coded: red = self-administered tickle, blue = experimenter-administered tickle. The graph shows normal “feeling of agency” in R, with lower intensity ratings in the self-administered tickle condition as compared to the experimenter-administered tickle condition (the scale goes from 0, “not ticklish at all,” to 10, “extremely ticklish”). B. Self-agency judgment task results. Mean subjective ratings of control over the movement of the blue box (from 0 = “no control” to 100 = “completely in control”), plotted as a function of objective variation in control (i.e., “noise” which is the proportion of time not in control during a trial). The group mean for each noise condition for healthy comparison participants are represented in gray, with error bars corresponding to 2 standard deviations from the mean. R's mean ratings are represented in red. For all participants, including R, the sense of control parametrically decreased as noise (i.e., objectively manipulated lack of control) increased. In all conditions, R's sense of self-agency was entirely within normal limits. Error bars = 2 standard deviations from the mean. Red = R's ratings. Gray = group mean of the healthy comparison participants.

R displayed the expected attenuation in response to tactile stimulation for the self-administered tickle (Figure 7a). R consistently reported higher tickle sensation ratings for experimenter-administered tickle as compared with self-administered tickle for all body parts (except for the knees, in which he reported no tickle sensation across both conditions). Of the 12 stimulation trials, there were only 3 trials (feet, armpits, and sides) where R was observed displaying signs of laughter and all occurred during the experimenter-administered tickle. The laughter was found to be most intense during the feet stimulation trial. When the experimenter tickled his feet, R laughed out loud while his entire body jerked back and forth and he exclaimed, “Yep, I definitely have sensation of it! Wow!” In contrast, after tickling his own feet he said, “Not as much. Not much.” Of note, R was never observed laughing or displaying jerking movements during any of the self-administered tickling trials. Given the asymmetrical damage of R's right hemisphere, it was conceivable that he might demonstrate a laterality effect whereby the tickle sensation is reduced (for both self-administered and experimenter-administered tickle) on the left side of his body (due to the contralateral representation of the body in the brain). For this reason, on the following day, the bottom of R's left and right feet were tickled separately. As before, R was found to be highly ticklish on both feet. Interestingly, laughter was only produced during the left foot stimulation, whereas jerking movements were observed during stimulation of either foot. When asked to rate the intensity of the tickle sensation, he rated the left foot as an 8 and the right foot as a 6, both ratings being slightly lower than his rating from the previous day when both feet were tickled simultaneously.

#### Core SA: Basic Self-Agency Tasks, Self-Agency Judgment Task

R's performance on the self-agency judgment task was indistinguishable from that of healthy comparison participants, across all levels of perceived control (Figure 7b). R's intact self-agency is represented clearly in Figure 7b where the graph displays a parametric relationship between his subjective level of perceived control and the objective proportion of time he was actually in control. Thus he was able to discriminate between actions under his voluntary control and actions not under his control.

#### Extended and Introspective SA: Basic Self-Concept tasks, Self-Consciousness Scale Revised (SCSR)

On the SCSR, R's score was within normal limits (i.e., within ±1 SD) on the public self-consciousness subscale [R = 12; normative mean (SD) = 13.5 (4.2)], the private self-consciousness subscale [R = 11; normative mean (SD) = 15.5 (4.8)], and social anxiety subscale [R = 13; normative mean (SD) = 8.8 (4.3)].

#### Extended and Introspective SA: Basic Self-Concept tasks, Self-Related Positivity Bias Task

**Figure 8:**
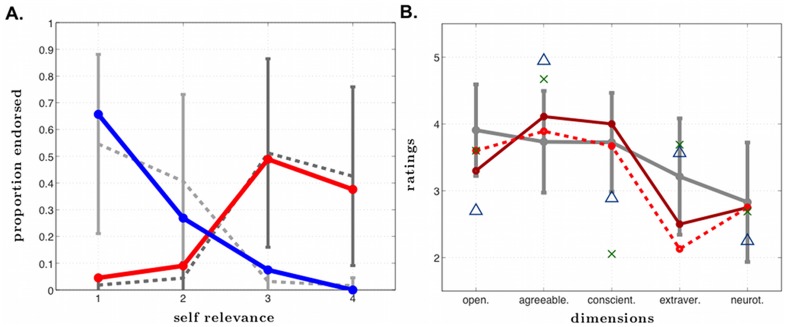
Self-concept. A. Positivity bias. Proportion of positive (red) and negative (blue) personality traits endorsed as a function of self-relevance ratings (from 1 = “not at all like me” to 4 = “very much like me”). The solid lines represent R's ratings and the dashed lines represent ratings from the comparison participants. Error bars represent one standard deviation. R's self-relevance rating curves are entirely within the normal range compared to other men of his age. B. Big Five Inventory ratings. R baseline: R's personality ratings of himself at initial assessment. R 1 year: R's personality ratings of himself one year later. Healthy male comparisons: average ratings for 796 healthy males in their 50's (error bars represent one standard deviation from the mean) [93]. Blue triangles: ratings of R by his mother. Green X's: ratings of R by his sister. *Note, we acknowledge we are connecting discrete data (not continuous data) with lines in this graph.

R demonstrated a positivity bias, following the typical pattern, when judging the degree to which personality traits were descriptive of him. He rated a greater percentage of positive traits as highly self-relevant (“very much like me”, 38%) than as not at all self-relevant (“not at all like me”, 5%), and a greater percentage of negative traits as not at all self-relevant (66%) than highly self-relevant (0%). His self-relevance rating curves were entirely within normal limits when compared to other men of his age (Figure 8a).

#### Extended and Introspective SA: Basic Self-Concept tasks, Big Five Inventory (BFI)

R's personality and self-concept ratings on the BFI [92] were highly stable across time, both at baseline and at the 1-year follow-up (Figure 8b). His combined item by item test-retest reliability was actually higher than a group of 53 healthy subjects who completed the same questionnaire using the same one-year retest interval (.75 versus .71) [95]. Interestingly, there were systematic discrepancies between the personality ratings that he provided, and those that his mother and sister independently provided about him. Whereas R viewed his level of agreeableness and conscientiousness to be within the average range, both his mother and his sister felt he was high on agreeableness and low on conscientiousness. Likewise, R rated himself as introverted whereas his mother and sister actually rated him as being more extraverted (a rating consistent with our own impressions based on our interactions with R over the past two decades). Taken together, these results suggest that R's self-concept is highly stable, but not entirely consistent with the way he is portraying himself to others.

#### Extended and Introspective SA: Anosognosia tasks, Modified Patient Competency Rating Scale (PCRS)

When assessing R's awareness for his condition, 8 of the 43 items revealed high discrepancy scores (i.e., scores where the average of the clinician ratings minus the average of R's ratings exceeded two points). R was most unaware of his smell deficit (discrepancy = −3.75) and taste deficit (discrepancy = −3.5), showing no knowledge that he had any deficits in these two sensory domains. When asked to comment on whether his ability to smell or taste has changed after his brain injury, he said, “I think my senses of taste and smell are quite close to normal… my sense of smell operates quite well and sufficiently.” Interestingly, R did not completely lack awareness for his memory deficits. For example, when R was asked in the SA interview, “What is it like to be you?”, he stated, “I'm a normal person with a bad memory.” When asked to write about any problems he has noticed with his memory since his brain injury he said, “I have trouble remembering small normal things that shouldn't be forgotten at all.” Despite his intact awareness of some memory deficits, he never rated his memory problems on the self-report assessments as extremely severe, suggesting he fails to appreciate the significance of his memory-related problems. In fact, 5 out of the 8 items on the PCRS with the greatest discrepancy scores (average discrepancy = −2.6) were related to memory (e.g., How much of a problem do I have in remembering important things I must do?). Together, this suggests that his lack of awareness for his memory impairment is better classified as anosodiaphoria (a lack of concern or understanding) rather than pure anosognosia (a complete lack of awareness).

#### Extended and Introspective SA: Anosognosia tasks, Smell Test

During all blindfolded trials, R was unable to smell the items and could not differentiate between scented versus non-scented items. While the blindfold was on, he was observed taking deep inhalations through his nose while commenting, “Nope, I don't smell much.” R's anosognosia was never reversed, despite direct exposure to his impairment once he removed the blindfold and immediately saw the item he was trying to smell. As soon as the blindfold was removed, he would name the item in front of him and state that he could smell it without any difficulty (in the case of the lemon and onion). For instance, when his blindfold was removed just after he had been presented with a lemon (and he failed to identify the stimulus), he reported that he could indeed smell the lemon. When he was explicitly asked why he couldn't smell the lemon with the blindfold on, he stated, “I guess sight makes things easier.” For the non-scented items, he reported that they were either odorless (tape) or had a smell that he could not recognize (pipe cleaner). On a separate occasion, a researcher asked R, “What if I told you that you lost your sense of smell and taste after your brain injury?” R quickly replied, “I wouldn't believe you. Nope, I can still taste and smell fine.” Thus R appears to be invariably unaware of his anosmia, and in all likelihood, his ageusia as well.

#### Extended and Introspective SA: Metacognitive and Conceptual Processing, SA interview

During the SA interview, R demonstrated an astute ability to introspect, even in response to abstract questions (see Table 1). Both his conceptual understanding and his ability to self-reflect indicate that he maintains preserved aspects of both Extended and Introspective SA. In Table 1 we report some relevant discourse samples from a two-hour interview.

**Table 1:** Self-awareness interview.

**Basic understanding of concepts**
**Int:** How would you define consciousness?
**R:** The body's awareness and reaction to what's going on in the environment around it.
**Int:** What do you think it would be like to be unconscious?
**R:** It wouldn't be anything, like anything you'd know about.
**Int:** Do you think it's possible that there are people out there that are unconscious?
**R:** Probably in hospitals.
**Int:** How would you define free will?
**R:** Choice to do things you would like to do… your own choice.
**Int:** How would you define the self?
**R:** Personal characteristics of a person that that person feels… their own personality in life, maybe.
**Int:** How would you define self-image?
**R:** Your idea of where your position is in society or life… Your impression, your thoughts of how you fit.
**Int:** How would you describe selfishness?
**R:** Trying to take too much from different places or people, or not sharing with others.
**Int:** Do you think the sense of self is like a concept?
**R:** Yes… it's an idea in your brain.
**Int:** Do you think the sense of self is like a memory?
**R:** Maybe, yeah… if the memory adds up to building a sense of self.
**Int:** Do you think that an accurate sense of self depends on having an accurate memory?
**R:** It might, some.
**Int:** Do you think that the sense of self depends on the existence of the body?
**R:** No, ‘cause you can lose different parts of the body and you're still the same.
**Int:** Okay. How much of your body do you think you would have to lose in order to lose your sense of self?
**R:** The brain.
**Int:** Do any animals other than humans have self-awareness?
**R:** Maybe apes… maybe dolphins, like Flipper.
**Int:** How would you define emotion?
**R:** A feeling you have. A deep, deep feeling, usually … love or hate or disgust.
**Int:** How would you define loneliness?
**R:** Sadness because of being all alone.
**Int:** How would you define sympathy?
**R:** Someone feeling sad for someone else because they were feeling sad or alone.
**Int:** How would you define compassion
**R:** A feeling for someone else … your love for others.
**Introspective questions**
**Int:** Do you have a sense of self?
**R:** Yes I do.
**Int:** How do you know that you have a sense of self?
**R:** I know who I am, what I need, how to survive … I'm surviving and thriving.
**Int:** Would you say that you are aware of yourself right now?
**R:** I think so, yep.
**Int:** Why do you think that you're aware of yourself right now?
**R:** I mean, you asked about “me” a lot, so that's been on my mind. You should be aware of what you're thinking… you're talking to more than just an empty chair [laughing].
**Int:** Do you think you can doubt your own existence?
**R:** No… you should always realize you are alive and existing.
**Int:** What if I told you that you weren't here right now?
**R:** I'd say you'd gone blind and deaf.
**Int:** How would it make you feel?
**R:** I would know that I am.
**Int:** How would it make you feel?
**R:** Like you're not telling the truth.
**Int:** If you had to describe to somebody what it is like to be you, what would you say?
**R:** Normal person with a bad memory… diabetic.
**Int:** What if I were to tell you that you were 200 years old?
**R:** I've missed a lot more than what I missed in this loss of memory [laughing].
**Int:** Do you feel like you always have a sense of self when you're awake?
**R:** Yeah, I think so, yeah.
**Int:** Why would you always have a sense of self when you're awake?
**R:** Look out for your best interest… for survival.
**Int:** Do you think you have a sense of self when you're asleep?
**R:** Maybe a little bit. I'm not sure what I think when I'm asleep.
**Int:** Do you think that the person you were yesterday when you woke up in the morning is the same person you were when you woke up this morning?
**R:** Yes.
**Int:** Is there anything mentally that changes, do you think?
**R:** Yeah. Knowledge in your brain. It affects opinion, ideas, every day is different … it's more than just a different date on the calendar.
**Int:** Do you think that there are things we do not know about ourselves?
**R:** Sure.
**Int:** What kinds of things would we not know about ourselves?
**R:** I don't know. [Laughing] How can I tell you what I don't know?
**Int:** Do you think that other people necessarily think of you the same way you think of yourself?
**R:** No, probably nobody.
**Int:** Why do you think that?
**R:** Because every brain has different thoughts.
**Int:** Do you think that other people can control your thoughts?
**R:** No.
**Int:** And why do you think that's not possible?
**R:** You control your own mind, hopefully.
**Int:** What if I were to tell you that your mind is the mind of somebody else?
**R:** When was the transplant? [Laughing] Brain transplant.
**Int:** What if I were to tell you I know you better than you know yourself?
**R:** I would think you're wrong.
**Int:** What if I were to tell you that you are aware that I'm aware?
**R:** I'd say you're right.
**Int:** You're aware that I'm aware?
**R:** I'm aware that you're aware that I'm aware.
**Int:** Oh, that was the next question.
**Int:** What if I were to tell you you were immortal, you'll live forever?
**R:** I'd say, if you can prove it to me, I'd love it.
**Int:** Are you scared to die?
**R:** I'd delay it as long as I can.
**Int:** What do you think happens after you die?
**R:** I just go… I don't really know? Just disappear.
**Int:** Imagine what it would be like to undergo immense suffering. Could you describe that in words?
**R:** It might be suffering beyond words, which means there's no words for it… ache and cry and all that.
**Int:** Do you have any feelings associated with imagining that?
**R:** Sore muscles… immense suffering would be like a giraffe with a sore throat or the centipede with athlete's foot without any things to scratch.

### Observations on R's behavior

As noted earlier, we indicated in the previous case report of R that “most people who meet R for the first time have no idea that anything is wrong. They see a normal-looking middle-aged man who walks, talks, listens, and acts no differently than the average person.” ([60], p. 104). These observations have been supplemented and supported by the wide range of experimental tasks carried out in the current investigation and underscore an important point: R manifests no readily observable disruptions in consciousness or SA. In fact, his behaviors often show a remarkable degree of depth and self-insight. For example, during the tickle test, when the experimenter asked permission to tickle his armpits, R somewhat jokingly replied, “Got a towel?” His humorous concern about perspiration was an indication that he was aware that he was sweating and he was also aware that the experimenter might feel it. Moreover, this “warning” indicates some intact ability to take another's perspective (theory of mind), as the presence of sweat could have made the experimenter uncomfortable. R's ability to tell jokes or make humorous comments spontaneously and wittingly is yet another indication of his preserved SA. Not only can he appreciate humor, but he also has a sophisticated understanding of what others will find humorous. Moreover, he caters his choice of jokes to the audience at hand. For example, around men he has been known to tell “dirty jokes” but he has never deployed such humor in the presence of women. Such behaviors emphasize that not only does R have preserved SA, but he also demonstrates an acute awareness for other people in his social environment.

## Discussion

The findings in this study confirm that R's SA is largely preserved. In keeping with previous work [60], [96], the findings show that R is a conscious, self-aware, and sentient human being despite the widespread destruction of cortical regions purported to play a critical role in SA, namely the insula, anterior cingulate cortex, and medial prefrontal cortex.

### Preserved aspects of Core SA

R has a largely preserved ability for self-recognition. He passed standard tests of basic self-recognition, including the mirror test, and was able to recognize himself in photographs (with contextual information) with 100% accuracy. He also showed a clear sense of self-agency, as assessed by both implicit measures (tickle test) and explicit measures (self-agency judgment task). We also showed in a previous study that he has preserved interoceptive awareness of cardiac sensations [96].

### Preserved aspects of Extended and Introspective SA

R exhibited a largely preserved self-concept, including a normal positivity bias and highly stable ratings of his personality across time. His general appreciation of his memory deficit indicates that, at the very least, he has some awareness for his cognitive limitations. He also displayed clear self-referential and introspective conceptual abilities, apparent most notably in his answers to the SA interview, but also in his preserved self-consciousness in social settings and on self-report questionnaires.

### Disrupted aspects of SA

Due to R's pervasive amnesia, his Extended SA, especially with regard to his autobiographical self [3], appears to be largely limited to the repositories of stored episodic and semantic knowledge consolidated during his childhood and young adult life. Consequently, according to his family, R's current view of himself is somewhat closer to the more introverted and reserved R that they knew before his brain damage (Figure 8b). Predictably, because of his anterograde amnesia, R has not fully updated the fact that he has become rather outgoing and extraverted following the onset of his brain damage. These results suggest the possibility that each time R thinks about who he is, he returns to a highly stable image of himself from the past. This is likely to be a result of the profound anterograde amnesia that has limited his ability to update his autobiographical self. Consequently, aspects of R's self-concept may be rather rigid and inflexible.

R's self-recognition from uncropped and context-embedded pictures of his face was perfect. However, R was able to recognize himself in only 50% of the pictures when the pictures of his face were cropped to exclude extra-facial information (Figure 6c). One should note that in healthy, non-brain injured individuals manipulations in the presentation of facial features and viewpoints (e.g., altered spatial relations, face inversion, profile viewpoints) can significantly affect face recognition [97], [98] and self-recognition [99]. Moreover, the defect might also reflect a difficulty for R in flexibly processing his self-image and adapting to unexpected changes. For example, there was a picture of him that he first rated as unfamiliar and then he reversed his answer and stated, “Oh that's me…but I have a different smile and they shaved my head for that picture.” Likewise, he frequently commented on the features that were different from his current appearance (e.g., “It's me, but it's been a few years since I've had a mustache”). Finally, R has extensive damage to the right temporal lobe, and to some extent, the left temporal lobe, in both gray and white matter, and this could cause some of his difficulties with face recognition. However, his perception of faces is intact and he performs within the average range on the Benton Facial Recognition Test, a neuropsychological measure which assesses one's ability to make fine-grained perceptual judgments about different faces that lack both context and extra-facial features [60].

R also had anosognosia for his anosmia. There are other reports of patients with anosognosia for anosmia following herpes simplex encephalitis [100], [101]. Although it is difficult to attribute the deficit in awareness to a specific brain region, it can be noted that in herpes simplex encephalitis, the damage often destroys the entire olfactory apparatus. Conversely, during loss of smell due to a bad cold or stemming from traumatic injury of the olfactory nerve, the peripheral input is cut off but the central processors remain intact. In R, the central comparator is gone, possibly precluding the automatic “awareness” of the loss. Perhaps this condition can be likened to Anton-Babinski syndrome whereby bilateral damage to the primary visual cortices renders a patient cortically blind, yet the patient adamantly claims to have normal vision.

When explicitly confronted with his anosmia during the smell test, R persisted in denying the impairment. Although he was unable to perceive the scent of a lemon with a blindfold on, he claimed that with the blindfold off he was able to smell it. When further queried, he proposed that this might be due to the aid of vision. The most plausible explanation is that the vision of a lemon triggered in him memories of the situation of smelling lemon scents, leading him to make an illusory attribution. It is a confabulation of sorts except that it is prompted by a logically related non-confabulatory percept. Nevertheless, it is clear that R has failed to incorporate the knowledge about his missing sense of smell (and taste) into his repository of self-related knowledge, even though he was able to incorporate some knowledge about his memory impairment.

### Implications for the different theoretical frameworks

Based on these results, we find little support for the hypotheses that implicate the insular cortex as critical to all aspects of SA [29], and the ACC as critical to Core SA and Introspective SA [18], [21], [33]. Likewise, hypotheses suggesting that the insula and ACC, in particular on the right, would be critical in facial self-recognition [19], are not supported.

The hypothesis that concerns the critical and unique role of the insular cortex in SA [20], [29] has been previously questioned in another experiment that demonstrated preserved interoceptive awareness of heartbeat sensations (a measure of Core SA) in R [96]. The presence of small patches of possibly functional cortical tissue in R's left anterior insula and ACC has been used to criticize the interpretation of the previous results [102]. However, in the context of the previous study [96], it is apparent that these tissues were not sufficient to support interoceptive awareness in R, since he lost, contrary to the comparison participants, all ability to report heart beat sensations under high-doses of isoproterenol, but only when alternative somatosensory pathways were blocked by anesthesia of the skin. Also, the new imaging results presented in the current study (including FLAIR, T2, DTI, and functional connectivity) show that these small regions of tissue within the left anterior insula and ACC are highly abnormal and have been largely disconnected (both anatomically and functionally) from the rest of the brain. Moreover, our volumetric estimates revealed only 10% of tissue remaining in his insula and 1% of tissue remaining in his ACC. It seems implausible that a phenomenon as complex as SA could be supported by such small patches of largely disconnected and defective neural tissue. In all likelihood, there are multiple pathways and body maps involved in SA [96], [103]. Additionally, we know of another herpes simplex encephalitis patient with complete bilateral destruction of the insular cortex who was also sentient and self-aware, whose case was recently published in a retrospective report [104]. Thus the hypothesis that the insular cortex is essential for SA [20], [29] is untenable. We also note that contrary to the proposed role of the insula in tickle sensation [29], R was able to experience and report feeling ticklish on both sides of his body.

Progressive degeneration of the anterior insula has been found in patients with frontotemporal dementia (FTD), which may contribute to FTD-related impairments in self-monitoring and social and emotional functioning [105], [106]. However, it is important to note that the complete loss of SA in FTD (referred to by [15]) is typically only found during the later stages of the degenerative process. By this point, the disease has spread to brain regions well beyond the insular cortex, raising difficulties for the conclusion that the loss of SA is caused by insular pathology.

The hypothesis that holds that the mPFC is critical for Extended and Introspective aspects of SA, including self-referential processing [1], [21], [22], [45], [46], can be questioned, at least when taken in an all-encompassing way, as central aspects of R's self-referential processing are largely preserved, as is his ability to introspect. However, some anatomical ambiguity remains, as the bilateral damage of R's mPFC was asymmetric, with substantially greater injury to the right hemisphere. While his self-referential processing was relatively preserved, R did appear to show deficits in the interaction between self and memory processes. In a previous study, we found that right mPFC damage impaired the normal enhancement of memory for self-relevant information (i.e., personality traits), known as the self-reference effect (SRE) [47]. Thus, based on the extent of R's mPFC damage, we would expect him to show similar deficits in the SRE. As mentioned above, R was impaired at updating his self-concept with new self-relevant information about his personality traits (e.g., extroversion). These findings remain compatible with the hypothesis that the mPFC facilitates memory for self-relevant information [47], [107], and at least in this sense, plays a role in self-referential processing and some aspects of extended SA.

By contrast, the hypothesis implicating brainstem nuclei in generating the “primordial feelings” essential to Core SA is entirely compatible with our findings (as R does not have brainstem damage), and is in keeping with the fact that selective damage to brainstem tegmentum has long been associated with impaired consciousness (see [8], [108] for relevant discussion). Furthermore, this hypothesis is also consistent with the striking presence of core SA and basic emotional functioning found in children who are missing their cortex due to hydranencephaly [8], [109].

Intact regions of R's cerebral cortex, such as the posteromedial cortex (which includes the posterior cingulate, the precuneus, and the retrosplenial cortices) and the inferior parietal lobule, could constitute the critical substrate for preserved SA in R. Both of these areas are critical nodes of the Default Mode Network. Activity in the posteromedial cortex, in particular, has been consistently associated with consciousness [110], [111] see also [8]). Of note, R had preserved functional connectivity within this region of the brain. Intracranial recordings have also associated the posteromedial cortex with self-referential processing [112]. Moreover, Dastjerdi and colleagues [112] found that intracranial electrodes placed near the retrosplenial cortices responded preferentially to autobiographical memory stimuli. These findings are consistent with neuropsychological research which has implicated the retrosplenial cortex in autobiographical memory retrieval [113], [114]. R's intact retrieval of some autobiographical knowledge might be mediated in part by the retrosplenial cortices. More generally, our findings are compatible with hypotheses invoking distributed neural systems as a substrate for SA and its components [24], [25], [26].

Given that R incurred his brain damage many years ago, it is reasonable to ask if the possibility of recovery of function by recruitment of other brain regions could explain his preserved SA. We simply do not know, and that is possible—however, based on the informal reports of caregivers soon after the brain injury (i.e., within the first two weeks of injury), basic aspects of R's SA already appeared intact during this early time period. Moreover, if other brain regions took over to maintain SA, the very possibility of recovery would imply that the damaged regions were not *necessary* for SA to operate.

### Conclusion

The evidence in the present study does not favor hypotheses that call for a critical and necessary role for the insula, ACC, and mPFC in producing the complex phenomenon of SA. Our findings are compatible with the hypothesis that SA is likely to emerge from distributed interactions among networks of brain regions that include the brainstem, thalamus, and posteromedial cortices. Finally, our findings emphasize the necessity of utilizing complementary methodologies, such as the human lesion method and functional neuroimaging, to aid in our understanding of causal brain-behavior relationships.
